# Primary Sclerosing Cholangitis Associated With Ulcerative Colitis Coexisting With Cholangiocarcinoma: A Case Report

**DOI:** 10.7759/cureus.62531

**Published:** 2024-06-17

**Authors:** Abdullgabbar M Hamid, Sultan A Alshoabi, Abdulkhaleq A Binnuhaid, Kamal Alsultan, Amel F Alzain, Abdulmannan M Aman

**Affiliations:** 1 Radiology, Rush University Medical Center, Chicago, USA; 2 Diagnostic Radiology Technology, College of Applied Medical Sciences, Taibah University, Al-Madinah Al-Munawwarah, SAU; 3 Radiology, Hadhramout University, Al Mukalla, YEM; 4 Medicine, University Medical Center, Taibah University, Al-Madinah al-Munawwarah, SAU

**Keywords:** multifocal bile duct strictures, beaded cholangiography appearance, cholangiocarcinoma, ulcerative colitis (uc), primary sclerosing cholangitis (psc)

## Abstract

Primary sclerosing cholangitis (PSC) is a rare chronic inflammatory disease in which multifocal fibrosis of bile ducts causes eventually narrowing and even blocking, forming multifocal strictures alternated to dilatations. Here, we reported an extremely rare case of PSC associated with ulcerative colitis (UC) and coexisting with cholangiocarcinoma in a 33-year-old male presented with right upper quadrant pain and dark urine. Liver function tests were deranged, and ERCP found a beaded cholangiography appearance due to multifocal bile duct strictures alternating with normal and dilated segments of the common hepatic duct and the intrahepatic bile ducts. We aim to document this typical case of PSC associated with UC and coexisted with cholangiocarcinoma to add the existing data on these rare pathologies.

## Introduction

Primary sclerosing cholangitis (PSC) is a chronic inflammatory disease characterized by multifocal fibrosis of intra- and/or extrahepatic bile ducts, which eventually causes narrowing and even blockage, leading to the formation of multifocal strictures alternating with dilatations [1,2]. PSC is very rare, affecting 0-16 people per 100,000, and ultimately leads to cirrhosis and end-stage liver disease [3]. It is an idiopathic disease closely associated with inflammatory bowel disease (IBD), particularly ulcerative colitis (UC) in 80% of patients, though the association between the gut and liver affection is unclear [2,4]. The second European Crohn’s and Colitis Organization (ECCO) evidence-based consensus on extraintestinal manifestations (EIMs) of IBD and anemia reports PSC as a classical EIM of IBD [5]. PSC coexists with autoimmune conditions in 25% of cases and is considered a premalignant condition, increasing the risk of cholangiocarcinoma and colorectal carcinoma. Three subtypes of PSC have been described: 1) classic, which affects both small and large bile ducts; 2) small ducts, which affect only small bile ducts; and 3) associated with autoimmune hepatitis, which affects both small and large bile ducts [6].

This case report aims to document PSC cases complicated by cholangiocarcinoma and highlight the role of medical imaging in diagnosing such rare cases. Moreover, a description of the endoscopic retrograde cholangiopancreatography (ERCP) and magnetic resonance imaging cholangiopancreatography (MRCP) features of PSC was done.

## Case presentation

A 33-year-old male presented with abdominal pain and was referred from an outside hospital to our medical center with suspected PSC. His symptoms began approximately three weeks prior, marked by dark urine and pain in the epigastric and right upper quadrant (RUQ) areas. He was taking Azathioprine (Imuran) for his UC. Blood tests revealed elevated liver enzymes, including alkaline phosphatase (ALP), alanine transaminase (ALT), and aspartate transaminase (AST). Despite discontinuing Imuran, his liver enzyme levels continued to rise.

**Figure 1:**
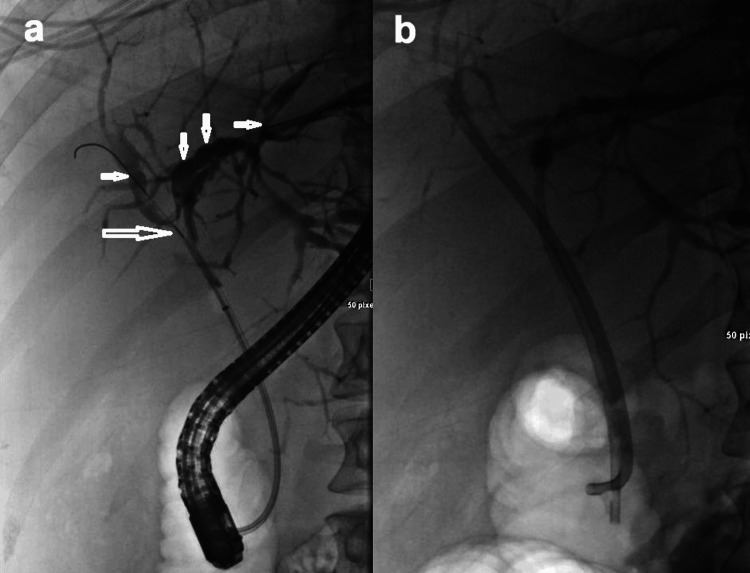
Selected images of cholangiogram showing a) a tight stricture (large arrow) in the CHD and additional multiple strictures (small arrows) in the left and right IHDs. b) Two stents were placed after dilatation of the areas of the strictures CHD: coronary heart disease; IHDs: ischemic heart diseases

The patient underwent an ERCP, which was challenging and required a needle knife papillotomy for cannulation. The cholangiogram revealed a normal caliber CBD with a tight stricture in the CHD and additional strictures in the left and right ischemic heart diseases (IHDs) (Figure 1a). Further injections were avoided due to concerns about potential cholangitis. Dilatation of the strictures in the CHD and IHDs was performed, resulting in some bile flow. Multiple biopsies from the strictures in the common, right, and left hepatic ducts showed inflammation but were negative for malignancy. Due to incomplete drainage and extensive manipulation, two 8 French, 12 cm stents were placed (Figure 1b). Some bile flow was observed. However, the patient continued to experience persistent pain after the procedure and was provided with hydration.

**Figure 2:**
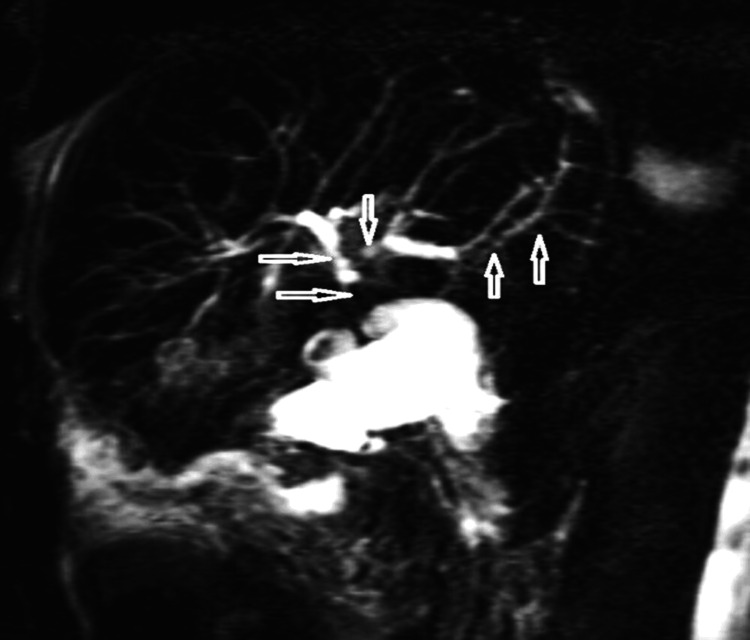
Selected image of T2 coronal HASTE radial MRCP showing sever stenosis of the hepatic duct and multiple areas of alternating IHD stenosis/dilatation (arrows) consistent with the diagnosis of PSC HASTE: half-Fourier acquisition single-shot turbo spin-echo; MRCP: magnetic resonance imaging cholangiopancreatography; IHD: ischemic heart disease; PSC: primary sclerosing cholangitis

In our hospital, a complete workup was performed. MRCP (Figure 2) demonstrated sever stenosis of the hepatic duct and multiple areas of alternating IHD stenosis/dilatation consistent with the diagnosis of PSC.

**Figure 3:**
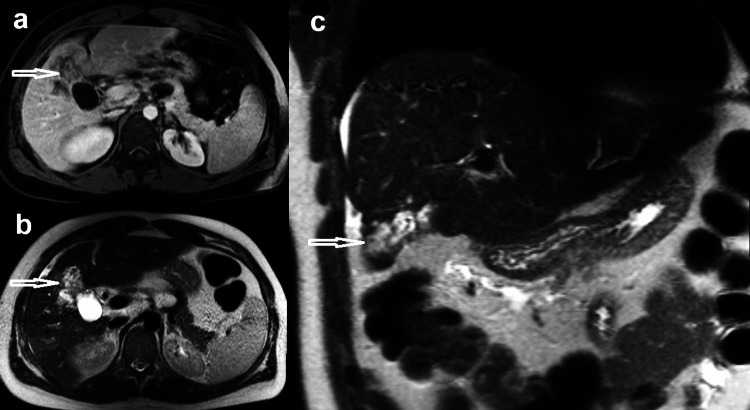
Selected images of magnetic resonance imaging showing a focal area of parenchymal atrophy with tubular hypointense area (arrow) on axial T1-weighted image (a), and hyperintense area (arrow) on axial T2-weighted image (b) in segment IV/V adjacent to the GB with mild enhancement (arrow) and retracted adjacent liver capsule appear on coronal section (c)

MRI showed a mild heterogeneous hepatic parenchyma. Focal area of parenchymal atrophy with tubular signal hypointense on T1-weighted images, and hyperintense on T2-weighted images in segment IV/V adjacent to the gallbladder (GB), associated with mild enhancement on post-infusion images and retracted adjacent liver capsule (Figure 3).

A random trans jugular liver biopsy (TJLB) was performed which showed cirrhotic liver (stage 4/4), in addition to portal tracts inflammation, ductular reaction, and cholestasis, compatible with clinical history of PSC.

**Figure 4:**
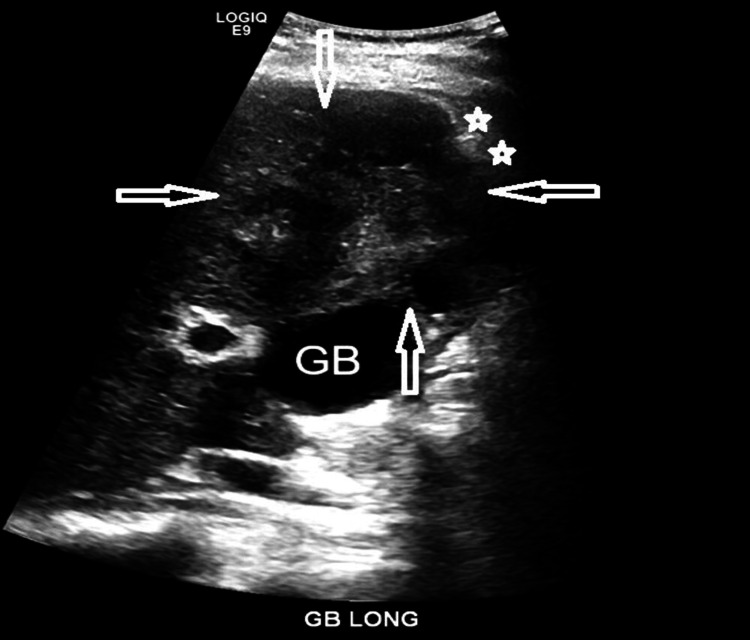
Ultrasonography image of the liver and gallbladder (GB) demonstrates a heterogeneous lesion (arrows) on segment IV/V as heterogeneous area with liver capsular retraction (stars)

**Figure 5:**
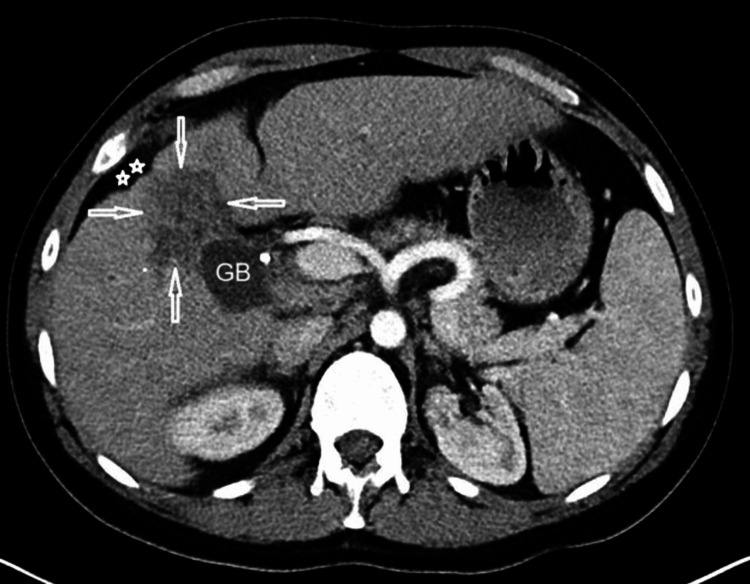
Selected contrast-enhanced computed tomography (CECT) image of the liver and gallbladder (GB) demonstrated the lesion (arrows) on segment IV as heterogeneous area with liver capsular retraction (stars)

Ultrasound (Figure 4) and CT (Figure 5) were performed and demonstrated the lesion on segment IV/V as a heterogeneous, predominantly hypoechoic area with capsular retraction. There was also mild thickening of the adjacent GB fundus.

Selected contrast-enhanced computed tomography (CECT) (Figure 5) images of the liver and GB demonstrated the lesion (arrows) on segment IV as a heterogeneous area with liver capsular retraction (stars).

Because of the suspicious appearance of the segment IV/V lesion, multiple tissue samples were taken from it under the US guidance and histopathology results returned as cholangiocarcinoma. The patient underwent radioembolization/Therasphere treatment.

**Figure 6:**
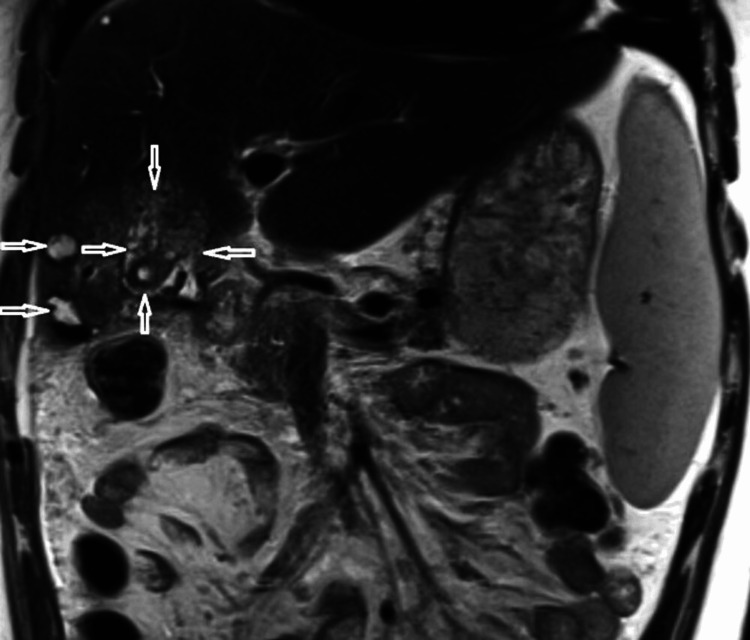
Selected coronal section of magnetic resonance imaging (T2) after stent and biopsy showing an enlargement of the T2 heterogeneous area on segment IV with multiple adjacent small abscesses (arrows)

Within the following six months, the patient was admitted many times to the hospital because of RUQ pain and fever concerning ascending cholangitis. Repeated MRI demonstrated an enlargement of the T2 heterogeneous area on segment IV/V with multiple adjacent small abscesses (Figure 6).

Selected coronal section of magnetic resonance imaging (T2) (Figure 6) after stent and biopsy showing an enlargement of the T2 heterogeneous area on segment IV with multiple adjacent small abscesses (arrows).

Ultimately, aspiration was performed many times. The patient continues to receive care at the same medical center.

## Discussion

PSC is a rare condition, and diagnosing it can be challenging, requiring accurate investigation as illustrated by this case report. PSC affects males more frequently than females at a ratio of 3:2, with a median age of onset of 41 years. The most common presenting symptoms are abdominal pain (20%), pruritus (10%), fatigue (6%), and jaundice (6%). The most frequent signs include hepatomegaly (44%) and splenomegaly (39%) [7]. Our patient, a 33-year-old male, presented with abdominal pain and hepatosplenomegaly. Elevated liver enzymes, such as AST and ALT in a cholestatic pattern, are hallmark findings of PSC. However, 30%-40% of patients have normal ALP [8]. In our case, all serum liver function tests, including ALP, were elevated along with dark urine.

PSC is a relatively common complication or EIM of UC, a diffuse inflammatory disorder of the colon [9]. It is estimated that roughly 70% of patients with PSC have underlying IBD, most frequently UC. Conversely, only approximately 5% of patients with UC will develop PSC. Our patient was a known case of UC under treatment when diagnosed with PSC. Cholangiocarcinoma, or bile duct carcinoma, is a malignant tumor originating from the cholangiocytes that line the bile ducts, and PSC is a risk factor in 10% of cases [10]. The incidence of cholangiocarcinoma increases 100-fold in patients with PSC. To improve survival rates, the American Association for the Study of Liver Diseases and the European Association for the Study of the Liver recommends annual imaging screenings for cholangiocarcinoma in adult PSC patients [11].

Regarding medical imaging, ultrasonography is the most widely available tool and is used as the first screening method for cholangiocarcinoma [12]. Focal capsular retraction and dilatation of the intrahepatic ducts are the main findings indicating the presence of a proximal tumor. However, its diagnostic value is limited by factors such as operator dependency, artifacts in the hepatobiliary system, and the isoechoic nature of the tumor [13]. In our patient, ultrasonography demonstrated a heterogeneous lesion near the GB with adjacent liver capsule retraction. CECT confirmed the presence of the lesion with liver capsule retraction. MRI/MRCP has greater diagnostic value in both diagnosing and monitoring patients suspected or known to have PSC. MRCP images can demonstrate the typical beading appearance of the intrahepatic and extrahepatic biliary ducts, suggesting the diagnosis. Due to its non-invasive nature, annual MRCP surveillance is recommended to monitor for cholangiocarcinoma, indicated by new filling defects within dilated biliary ducts or increased focal/segmental narrowing and dilatation of the proximal biliary ducts. Adding diffusion-weighted imaging (DWI) is valuable for diagnosing PSC and evaluating disease severity and fibrosis burden [14].

In the literature, ERCP is reported as the gold standard imaging method for diagnosing PSC [15]. Our patient underwent an ERCP, which revealed multiple strictures in the common hepatic duct (CHD) and intrahepatic bile ducts (IHBDs), a typical finding for PSC. PSC appears as a beaded cholangiographic pattern due to multifocal bile duct strictures alternating with normal or dilated bile duct segments. The bile duct strictures are usually short, annular, or band-like, and long confluent strictures may be found in advanced cases. Both extrahepatic and IHBD involvement is reported in 75% of patients [16].

Currently, there is no effective treatment to delay the progression of PSC or improve liver transplant-free survival [17]. In our patient, the coexistence of cholangiocarcinoma worsened the prognosis.

## Conclusions

PSC is an inflammatory disease that appears on MRCP/ERCP as a beaded cholangiography appearance due to multifocal bile duct strictures alternating with normal and dilated segments of the extra- and IHBD. PSC is an EIM of UC and a risk factor for cholangiocarcinoma with very rare coexistence. Annual MRCP surveillance is a suitable noninvasive tool to monitor the disease.

## References

[1] Cazzagon N, Sarcognato S, Catanzaro E, Bonaiuto E, Peviani M, Pezzato F, Motta R (2024). Primary sclerosing cholangitis: diagnostic criteria. Tomography.

[2] Karlsen TH, Folseraas T, Thorburn D, Vesterhus M (2017). Primary sclerosing cholangitis - a comprehensive review. J Hepatol.

[3] Lazaridis KN, LaRusso NF (2015). The cholangiopathies. Mayo Clin Proc.

[4] Manganis CD, Chapman RW, Culver EL (2020). Review of primary sclerosing cholangitis with increased IgG4 levels. World J Gastroenterol.

[5] Gordon H, Burisch J, Ellul P (2024). Ecco guidelines on extraintestinal manifestations in inflammatory bowel disease. J Crohns Colitis.

[6] Rawla P, Samant H (2024). Primary sclerosing cholangitis. StatPearls [Internet].

[7] Lazaridis KN, LaRusso NF (2016). Primary sclerosing cholangitis. N Engl J Med.

[8] Rabiee A, Silveira MG (2021). Primary sclerosing cholangitis. Transl Gastroenterol Hepatol.

[9] Yano K, Moroi R, Shiga H (2022). Analysis of the disease activity of ulcerative colitis with and without concomitant primary sclerosing cholangitis: an investigation using a nationwide database in Japan. JGH Open.

[10] Alsaleh M, Leftley Z, Barbera TA (2019). Cholangiocarcinoma: a guide for the nonspecialist. Int J Gen Med.

[11] Morgan MA, Khot R, Sundaram KM (2023). Primary sclerosing cholangitis: review for radiologists. Abdom Radiol (NY).

[12] Sungkasubun P, Siripongsakun S, Akkarachinorate K (2016). Ultrasound screening for cholangiocarcinoma could detect premalignant lesions and early-stage diseases with survival benefits: a population-based prospective study of 4,225 subjects in an endemic area. BMC Cancer.

[13] Alsaedi HI, Krsoom AM, Alshoabi SA, Alsharif WM (2022). Investigation study of ultrasound practitioners' awareness about artefacts of hepatobiliary imaging in almadinah Almunawwarah. Pak J Med Sci.

[14] Kovač JD, Ješić R, Stanisavljević D, Kovač B, Maksimovic R (2013). MR imaging of primary sclerosing cholangitis: additional value of diffusion-weighted imaging and ADC measurement. Acta Radiol.

[15] Tharian B, George NE, Tham TC (2015). What is the current role of endoscopy in primary sclerosing cholangitis?. World J Gastrointest Endosc.

[16] Mohammad Alizadeh AH, Shahnazi A, Rasoulzadeh A, Shams E, Mohammadi M, Darabi F, Behdad M (2012). Characteristic findings of primary sclerosing cholangitis on endoscopic retrograde cholangiography: which is the most common finding?. Clin Med Insights Gastroenterol.

[17] Floreani A, De Martin S (2021). Treatment of primary sclerosing cholangitis. Dig Liver Dis.

